# 

**Published:** 1890-01

**Authors:** 


THE Medical Society of the County of Erie will hold its regular annual meeting on Tuesday, January 14, 1890, at 10 o'clock A. M. in the lecture room of the Y. M. C. A., corner West Mohawk and Pearl streets, under the presidency of Dr. R. L. Banta, of this city.

---

THE eighty-fourth annual meeting of the Medical Society of the State of New York will be held in the city of Albany Tuesday, Wednesday, and Thursday, February 4, 5, and 6, 1890, under the presidency of Dr. Daniel Lewis, of New York City.

### BOOKS AND PAMPHLETS RECEIVED.

A Treatise on Materia Medica, Pharmacology, and Therapeutics. By John V. Shoemaker, A. M., M. D., and John Aulde, M. D. In two volumes. Volume I. F. A. Davis, publisher, Philadelphia and London. 1889.

A Text-Book of Practical Medicine. Designed for the use of students and practitioners of medicine. By Alfred L. Loomis, M. D., LL. D. Eighth edition, revised and enlarged, with 215 illustrations. Pp. xvii., 1147. New York: William Wood & Co. 1889.

A Hand-Book of Pathological Anatomy and Histology; with an introductory lecture on Post Mortem Examinations, and the Methods of Preserving and Examining Diseased Tissues. By Francis Delafield, M. D., and T. Mitchell Prudden, M. D. Third edition. Illustrated. New York: William Wood & Co. 1889.

Transactions of the Association of American Physicians. Fourth volume. Philadelphia: Printed for the Association. 1889.

Oxygen and other Gases in Medicine and Surgery. By J. N. Demarquay, M. D. Translated, with Notes, Additions, and Omissions, by Samuel S. Wallian, A. M., M. D. Illustrated with fine wood engravings. Philadelphia and London: F. A. Davis, publisher. 1889.

Education and Culture as Related to the Health and Diseases of Women. By Alex. J. C. Skene, M. D. The Physician's Leisure Library. 1889. Geo. S. Davis, Detroit, Mich.

Diabetes. By A. H. Smith, M. D. The Physician's Leisure Library. 1889. Geo. S. Davis, Detroit, Mich.

Mittheilung aus der Kgl. Frauenklinik in Dresden. Zur Behandlung der Uterusruptur. Von Prof. Dr. Leopold. Leipzig. Druck von A. Th. Engelhardt. 1889.

Concealed Pregnancy: Its Relations to Abdominal Surgery. By Albert Vander Veer, M. D. Reprint from the *American Journal of Obstetrics and Diseases of Women and Children*, November, 1889. New York: William Wood & Co., publishers.

The Surgical Treatment of Erysipelas in Children. By A. Seibert, M. D. Reprint from the New York *Medical Journal* for October 19, 1889.

The Cure of Hernia by the Use of the Buried Antiseptic Animal Suture. By Henry O. Marcy, A. M., M. D., LL. D. Reprint from the *Journal of the American Medical Association*, November 2, 1889. Chicago.

Chronic Inversion of the Uterus. Reduction by a New Method. By Henry O. Marcy, A. M., M. D., LL. D. Reprint from the *Journal of the American Medical Association*, July 20, 1889. Chicago.

A Hitherto Undescribed Disease of the Ovary; Endothelioma changing to Angeioma and Hematoma. By Mary A. Dixon Jones, M. D., Brooklyn. Reprint from the New York *Medical Journal* for September 28, 1889.

Two Cases of Removal of Uterine Myoma: one, Suprapubic Hysterectomy; the other Complete Hysterectomy. By Mary A. Dixon Jones, M. D. Reprint from the New York *Medical Journal* for August 25 and September 1, 1888.

Case of Tuberculosis Papillomatosa Cutis, with Remarks on the Relation of Papilloma to Syphilis, Lupus, etc. Illustrated with chromo-lithograph plate. By Prince A. Morrow, A. M., M. D. Reprint from the *Journal of Cutaneous and Genito-Urinary Diseases*, October and November, 1888.

Personal Observations of Leprosy in Mexico and the Sandwich Islands. By Prince A. Morrow, A. M., M. D. Reprint from the New York *Medical Jonrnal* for July 27, 1889.

Proceedings and Addresses at a Sanitary Convention, held at Ludington, Mich., July 11 and 12, 1889. Under the Direction of a Committee of the State Board of Health and a Committee of Citizens of Ludington. Supplement to the Report of the Michigan State Board of Health for the Year 1890. Lansing: Darius D. Thorp, State Printer and Binder. 1889.

Annual Report of the Treasurer of the United States. 1889. Washington: Government Printing Office.

Cornell University, College of Agriculture. Bulletin of the Agricultural Experiment Station. Entomological Department. XI. November, 1889. On a Saw-Fly Borer in Wheat. Published by the University, Ithaca, N. Y.

The Identification of Criminals. Its Value as a Preventive of Crime; and the Importance of Unity of Action among Prison Officials in Securing a Fixed and General System. Read at the Congress of the National Prison Association, at Nashville, Tennessee, November 16-20, 1889. By Charles E. Felton, Superintendent of the House of Correction, Chicago, Ill. Knight & Leonard Co., Printers.

Thirty-Seventh Annual Announcement of the Medical Department, University of Vermont. For the Year 1890.

Monthly Bulletin of the New York State Board of Health. October, 1889.

Report of Deaths and Contagious Diseases for October and November, 1889. Newport, R. I.

Health in Michigan, November, 1889.

Weekly Abstract of Sanitary Reports. Vol. IV. Nos. 47, 48, 49, and 50.

---

### Literary and Other Notes.

A VALUABLE AND UNIQUE BUSINESS CALENDAR.—The most convenient, valuable, and unique business table or desk calendar, for 1890, is the Columbia Bicycle Calendar and Stand, issued by the Pope Mfg. Co., of Boston, Mass. The calendar proper is in the form of a pad containing 366 leaves, each 5½x2¾ in.; one for each day of the year, to be torn off daily, and one for the entire year. A portion of each leaf is left blank for memoranda, and as the leaves are not pasted but sewed at the end, any entire leaf can be exposed whenever desired. By an ingenious device the leaves tear off independently, leaving no stub. The pad rests upon a portable stand, containing pen-rack and pencil-holder, and when placed upon a desk or writing table the entire surface of the date leaf is brought directly, and left constantly, before the eye, furnishing date and memoranda impossible to be overlooked. The stand is made of colored wood, mounted with raised letters in brass, and is practically indestructible. The days of the week, the number of the days of the year past and to come are specified, and upon each slip appear quotations pertaining to cycling from leading publications and prominent writers; and, although this is the fifth year of the calendar, they are fresh and new, mentioning the notable events in cycling, opinions of medical authorities, clergymen, and other professional gentlemen, the rights of cyclers upon the road, cycling statistics, records, advice about costumes, directions for road making, and other interesting matter, the whole forming a virtual encyclopedia of cycling. Beside the cycling quotations there are many pertaining to typewriting, with occasional reference to the typewriters made by the Pope Mfg. Co. The information contained on the calendar, would, if placed in book type, make a fair-sized volume.

---

THE *Cosmopolitan* for January, 1890, will contain the following: Bouguereau, Artist and Man, illustrated, Carroll Beckwith; Columbia College, illustrated, Hjalmar Hjorth Boyesen; Thrones That Will

Totter Next, Mayo W. Hazeltine; Sugar Cane and Sugar Making, illustrated, William H. Ballou; Two Birds, poem, Frank Dempster Sherman; The Development of the Coat and Waistcoat, illustrated, Wm. Hamilton Bell; Had I Your Love, poem, M. C. Gillington; A Cruise Around Antigua, in the field papers, illustrated, Poultney Bigelow; Famous Beauties, illustrated, Elizabeth Bisland; Blenheim, the Famous, illustrated, Charles S. Pelham-Clinton; The Romantic Story of a Great Corporation, illustrated, J. Macdonald Oxley; St. Mary of the Angels, Thomas A. Janvier, illustrated by F. Hopkinson Smith, A. E. Sterner, Louis J. Rhead and from photographs; Ode to Sorrow, poem, William Bronson Le Duc; Social Problems, Edward Everett Hale; In the Library, illustrated, William S. Walsh, George Parsons Lathrop.

This magazine is fast achieving a reputation for excellence that will establish it in the forefront of American monthlies, and it should find a place in every household of culture and refinement.

In this connection we invite attention to the following:

COSMOPOLITAN MAGAZINE,
MADISON SQUARE BANK BUILDING,
Fifth Avenue, Broadway and Twenty-fifth street, New York.

*Dear Sir*—Please call attention to the clause in advertisement of your journal in combination with the *Cosmopolitan* magazine which makes the offer apply to "New Subscribers Only." The price of the *Cosmopolitan* is so low that it is impossible for us to receive renewals at anything less than the regular price of $2.40. The combination is intended to bring the magazine to the attention of those not already familiar with it. Please ask persons proposing to subscribe for the two journals in combination, if they are already subscribers to the *Cosmopolitan*. If so, it will save time and correspondence to have matters explained fully in the beginning.

Yours very truly,
THE COSMOPOLITAN.

DECEMBER 12, 1889.

---

THE PHILADELPHIA MEDICAL AND SURGICAL REPORTER presents an attractive offer on our advertising page that will well repay reading and acting upon. If any one should ask our opinion (which surely is not needed) we should answer that we regard the *Reporter* as well worth the full subscription price, $5.00 a year, and that it is one of the best of journals.

---

BOSTON'S new magazine, *The Transatlantic*, in its issue of December 14th, contains a fine portrait of Emile Zola; an extended extract (the first printed in this country) from his new novel, "The Human Beast"; a comparative criticism of his early work, just republished,

"Le Vœu d'une Morte"; and an interesting interview with Zola, in which he announces his candidacy for the French Academy. Other features of the number are Alphonse Daudet's preface to his new play, "The Struggle for Life," the reigning sensation on the Paris stage; a picturesque review of the Paris Exposition; two pages of music specially composed for a dance in "The Dead Heart," the play that Henry Irving is now running at the London Lyceum Theater; a novelette, "Figaro I. and Figaro II."; and many shorter articles of interest.

---

THE following letter exhibits such amazing effrontery that we publish it for the information of our exchanges:

J. H. CHAMBERS & Co., Publishers, 914 Locust street.

St. Louis, December 3, 1889.

BUFFALO MEDICAL AND SURGICAL JOURNAL, Buffalo, N. Y.:

*Gentlemen*—To answer the many requests which have been made by general medical journals to exchange with the *Annals of Surgery*, and whereas our editor has no use for such journals, we have concluded to mail the *Annals of Surgery* to such journals, with the understanding that they give editorial notices at least three times a year of the *Annals*.

Should you desire the *Annals of Surgery* during next year, kindly inform us, accepting of those conditions, and oblige,

Yours truly,

J. H. CHAMBERS & CO.

The BUFFALO MEDICAL AND SURGICAL JOURNAL has a high appreciation of the *Annals*, but we cannot offer it a premium to exchange with us. If the editor of the *Annals* has no use for the JOURNAL, we must, perforce, remain content, but we submit that we might be spared the painful duty of reminding the publishers, Messrs. J. H. Chambers & Co.—we cheerfully give the firm name in full—that there are some courtesies not more "honored in their breach."

---

THE NATIONAL MAGAZINE for January announces two new and valuable departments—"Biblical Literature" and "Pedagogy"—with Rev. J. C. Quinn, Ph. D., and J. S. Mills, A. M., President of Western College, as editors. Agricultural readers will be especially interested in the new "Institute of Agriculture," described in this number—a part of the University Extension System of the National University of Chicago, whose non-resident or correspondence undergraduate and post-graduate courses have met with such favor. Other articles are by Prof. E. A. Birge, of the University of Wisconsin, and eminent specialists. Published at 147 Throop street, Chicago, Ill. Subscription, $1.00 per year. Sample copy, 10 cents.

STATISTICS OF LEPROSY IN THE UNITED STATES.—In view of the general impression that Leprosy is spreading in this country, it is desirable, in the interest of the public health, to obtain accurate information upon this point. The undersigned is engaged in collecting statistics of all cases of leprosy in the United States, and he would ask members of the profession to aid in this work by sending a report of any case or cases under their observation, or coming within their knowledge.

Please give location, age, sex, and nationality of the patient, and the form of the disease—Tubercular or Anesthetic; also any facts bearing upon the question of contagion and heredity.

Address DR. PRINCE A. MORROW,
*Journal of Cutaneous and Genito-Urinary Diseases*,
66 West 40th street, New York.

---

THE Transactions of the American Association of Obstetricans and Gynecologists, Vol. II., 1889, is now ready. Orders should be addressed to the Association's printer, William J. Dornan, 100 North Seventh street, Philadelphia, Pa., or to the undersigned,

WILLIAM WARREN POTTER, M. D., Secretary,
284 Franklin st., Buffalo, N. Y.

---

MESSRS. WORTHINGTON & CO., publishers, 747 Broadway, New York, announce in their Fall list an exceedingly attractive catalogue of choice literature, under the name of the World Library. Each volume is printed in large, clear type, on good paper, illustrated and handsomely bound in black and gold. Price each, $1.00.

---

THE following notice appears in the *Journal of the American Medical Association*, December 21, 1889, and is reproduced for the information of all concerned:

AMERICAN MEDICAL ASSOCIATION, SECTION OF OBSTETRICS AND DISEASES OF WOMEN.

FORTY-FIRST ANNUAL MEETING.

The officers of the Section of Obstetrics and Diseases of Women respectfully request those who desire to read papers in that section at the meeting of the American Medical Association to be held in Nashville, Tenn., May 20–23, to communicate the titles thereof to either of the undersigned not later than January 15, 1890.

WILLIAM WARREN POTTER, *Chairman*,
284 Franklin street, Buffalo, N. Y.
JOSEPH HOFFMAN, *Secretary*,
126 W. Diamond street, Philadelphia, Pa.

